# Incidence and predictors of hyperglycemic emergencies among adult diabetic patients in Bahir Dar city public hospitals, Northwest Ethiopia, 2021: A multicenter retrospective follow-up study

**DOI:** 10.3389/fpubh.2023.1116713

**Published:** 2023-03-17

**Authors:** Melsew Dagne Abate, Ayele Semachew, Solomon Emishaw, Fentahun Meseret, Molla Azmeraw, Dawit Algaw, Dessie Temesgen, Sefineh Fenta Feleke, Ahmed Nuru, Makda Abate, Berihun Bantie, Atsedemariam Andualem

**Affiliations:** ^1^Department of Nursing, College of Health Sciences, Woldia University, Woldia, Ethiopia; ^2^Department of Adult Health Nursing, College of Medicine and Health Sciences, Bahir Dar University, Bahir Dar, Ethiopia; ^3^Department of Emergency and Critical Care Nursing, College of Medicine and Health Sciences, Bahir Dar University, Bahir Dar, Ethiopia; ^4^Department of Pediatrics and Child Health Nursing, College of Health and Medical Sciences, Haramaya University, Harar, Ethiopia; ^5^Department of Nursing, Bahirdar Health Sciences College, Bahir Dar, Ethiopia; ^6^Department of Public Health, College of Health Sciences, Woldia University, Woldia, Ethiopia; ^7^Department of Nursing, College of Medicine and Health Sciences, Wolkite University, Wolkite, Ethiopia; ^8^Department of Nursing, College of Medicine and Health Sciences, Debre Berhan University, Debre Berhan, Ethiopia; ^9^Department of Comprehensive Nursing, College of Health Science, Debre Tabor University, Debre Tabor, Ethiopia; ^10^Department of Nursing, School of Nursing and Midwifery, Injibara University, Injibara, Ethiopia

**Keywords:** hyperglycemic emergencies, incidence, adult diabetic patients, Ethiopia, predictors

## Abstract

**Background:**

Diabetic ketoacidosis and hyperglycemic hyperosmolar syndrome are the two commonly known life-threatening hyperglycemic emergencies of diabetes mellitus. Despite the growing hyperglycemic emergency impact among adult patients with diabetes, its incidence and predictors have not been well studied in Ethiopia. Thus, this study aimed to assess the incidence and predictors of hyperglycemic emergencies among adult patients with diabetes.

**Method:**

A retrospective follow-up study design was conducted among a randomly selected sample of 453 adult patients with diabetes. Data were entered into EPI data version 4.6 and analyzed using STATA version 14.0. A Cox-proportional hazard regression model was fitted to identify the independent predictors of hyperglycemic emergencies, and variables having a *p* < 0.05 in the multivariable model were considered statistically significant.

**Result:**

Among the total adult patients with diabetes included in the study, 147 (32.45%) developed hyperglycemic emergencies. Hence, the overall incidence of hyperglycemic emergencies was 14.6 per 100 person-years observation. The incidence of diabetic ketoacidosis was 12.5 per 100 person-years (35.6 and 6.3 among T1DM and T2DM, respectively). The incidence of the hyperglycemic hyperosmolar syndrome was 2.1 per 100 person-years (0.9 and 2.4 among T1DM and T2DM, respectively). The overall median free survival time was 53.85 months. Type 1 diabetes mellitus [AHR = 2.75, 95% CI (1.68, 4.51)], diabetes duration of ≥ 3 years [AHR = 0.33, 95% CI (0.21, 0.50)], recent acute illness [AHR = 2.99, 95% CI (2.03, 4.43)], presence of comorbidity [AHR = 2.36, 95% CI (1.53, 3.63)], poor glycemic control [AHR = 3.47, 95% CI (2.17, 5.56)], history of medication non-compliance [AHR = 1.85,95% CI (1.24, 2.76)], follow-up frequency of 2–3 months [AHR = 1.79,95% CI (1.06, 3.01)], and without community health insurance [AHR = 1.63, 95% CI (1.14, 2.35)] were significant predictors of hyperglycemic emergencies.

**Conclusion:**

The incidence of hyperglycemic emergencies was high. Therefore, giving greater attention to patients with identified predictors could decrease the occurrence of hyperglycemic emergencies and related public health and economic impacts.

## Background

Diabetes mellitus is a chronic metabolic disorder characterized by hyperglycemia associated with defects in insulin secretion, insulin action, or both (1). Diabetes mellitus is frequently associated with life-threatening hyperglycemic emergencies (2) that include diabetic ketoacidosis and hyperglycemic hyperosmolar syndrome (3).

Globally, diabetes prevalence and its impact have increased dramatically, particularly in sub-Saharan Africa (4, 5). Consequently, hyperglycemic emergencies (HGEs) continue to be a significant health and socioeconomic problem among adults with diabetes mellitus (DM) (6, 7).

According to a World Health Organization report (2016), there were 1.6 million diabetic-related deaths (8), and HGEs are the leading cause of diabetic-related morbidity, mortality, and healthcare costs among adults with diabetes (9). In the United States, the Centers for Disease Control and Prevention (CDC) report showed that the rate of hospitalization due to HGEs steadily increased with an annual hospitalization rate of 9.7 per 1,000 adults with diabetes (7). Whereas, in Africa, HGEs account for more than 12% of diabetic-related admissions (10, 11). Worldwide, different studies showed that the mortality rate ranges from 2 to 5% for diabetic ketoacidosis (DKA) and 10–20% for the hyperglycemic hyperosmolar syndrome (HHS) (12, 13), with higher premature mortality in Sub-Saharan Africa including Ethiopia (4, 14). HGEs' burden constitutes a healthcare cost crisis for patients and the national economy of the country (9, 15). In Ethiopia, hyperglycemic emergencies take a significant part in adult medical admission and the cost of inpatient diabetes management both in drug use and bed occupancy (16, 17).

A systematic review study conducted in different regions of the world revealed that the incidence of DKA ranges from 0 to 263 per 1,000 persons per year, with the highest incidence rate in China (263 per 1,000 person-years) and the lowest incidence rate in Israel and North America (0 events per 1,000 person-year) (18). Even though the exact incidence rate of HHS is not known, it is estimated to account for < 1% of diabetic-related hospital admissions among patients with diabetes (7, 19). A study showed that the incidence of hyperglycemic emergencies was 7.46% in Thailand (20) and the incidence has significantly increased in the USA (6, 21). Whereas, in Korea, hospitalization incidence rates increased from 1.78 per 1,000 diabetic cases in 2004 to 2.15 per 1,000 diabetic cases in 2013 and the predicted hospitalization incidence rate will increase by 2030 (22). A study in Nigeria (23) revealed 40% of hospitalization incidence, while a study in Ethiopia showed 38.2% of hospitalization from all DM-related admissions (24).

In sub-Saharan countries, lack of funding for non-communicable diseases, poor public healthcare, lack of proper health education, poor self-glycemic control, and lack of availability of studies and guidelines specific to the population have a great impact on the rising HGEs burden (4, 25). Its incidence could be affected and vary with socio-demographic, treatment, and clinical-related factors (14, 26), and the risk factors varied across the findings of previous studies (27–29).

Despite the increasing worldwide burden of diabetes and HGEs, data on the incidence among known adult patients with diabetes are limited, particularly in Africa, and the exact incidence of HHS is not identified worldwide (19). Previous studies revealed scarce data regarding the epidemiology of HGEs and recommend further studies on their incidences (18).

In Ethiopia, limited studies have been conducted and assessed the prevalence and associated factors of acute complications of DM (24, 29, 30). However, these studies did not address the incidence of HGEs among known adult patients with diabetes, and the predictors were not well addressed. No study was conducted to show the exact incidence of HHS and the time to develop HGEs among adult patients with diabetes. There is a study regarding the incidence and predictors of DKA among children with diabetes (26). However, as per our search technique, there is no study conducted in Ethiopia on the incidence and predictors of HGEs (DKA and HHS) among adults diagnosed with DM. Therefore, this study aims to identify the incidence, predictors, and time duration to develop HGEs among adults diagnosed with diabetes. In addition, it aims to show the exact incidence of HHS.

## Methods and materials

### Study area and period

This study was conducted in Bahir Dar public hospitals, North West Ethiopia from 1 January 2016 to 31 December 2020, and data were collected from 16 March 2021 to 14 April 2021. Bahir Dar is the capital city of Amhara Regional State, which is 565 km away from Addis Ababa, the capital city of Ethiopia. Currently, in the city, there are three public hospitals, namely, Felege Hiwot Comprehensive Specialized Hospital (FHCSH), Tibebe Ghion Specialized Teaching Hospital (TGSTH), and Addis Alem Primary Hospital (AAPH). Chronic disease services, including diabetes mellitus treatment and follow-up services, are being delivered in all these public hospitals.

Felege Hiwot Hospital is one of the compressive specialized public hospitals, which has a 500 bed capacity and around 15 adult outpatient departments (OPD) serving about 5 million people coming from more than 500 km distance. Tibebe Ghion Specialized Referral Hospital is a teaching hospital that has a 459 bed capacity and around 14 outpatient departments serving more than 5 million people. Addis Alem Hospital is a primary hospital with 47 bed occupancy and eight outpatient departments serving 100,000 people.

#### Study design

This is a retrospective follow-up study.

### Population

#### Source population

All adults (age greater than or equal to 18 years old) diagnosed with T1DM and T2DM, and who had chronic diabetes mellitus follow-up in Bahir Dar city public hospitals.

#### Study population

All adults diagnosed with T1DM and T2DM and who have DM followed-up from 1 January 2016 to 31 December 2020.

### Eligibility criteria

#### Inclusion criteria

All adults whose age ≥18 years old and diagnosed with T1DM and T2DM had a DM follow-up from 1 January 2016 to 31 December 2020.

#### Exclusion criteria

All individuals who developed HGEs during the first diagnosis of DM and whose records are incomplete for important variables (date of diagnosis of DM and date of the event or last observation) and did not have at least one follow-up after the first diagnosis were excluded from the study.

### Sample size and sampling procedure

#### Sample size determination

For sample size determination, the Stat Calc function of Epi Info software version 7 was used by applying the cohort sample size calculation technique and taking common predictor variables for HGEs with 95% confidence interval, 80% power, and a 1:1 ratio of unexposed to exposed patients. The predictor variables were glycemic control (30), recent acute illness (31), missed insulin dose (31), presence of comorbidity (32), and type of DM (32). From these predictor variables, we obtained a maximum sample size for the type of DM (T1DM) with the proportion of exposed (33.1%) and non-exposed (21.1%) patients. Finally, the maximum sample size of the study was 473 after a 10% non-respondent rate had been added.

#### Sampling techniques and procedures

All public hospitals in Bahir Dar city (Addis Alem primary hospital, Tibebe Ghion, and Felege Hiwot Comprehensive Specialized Hospital) were selected. There were about 1542, 500, and 800 adults diagnosed with DM in Felege Hiwot, Tibebe Ghion, and Addis Alem, respectively, with a total of 2842 adult patients with diabetes between 1 January 2016 and 31 December 2020. Then, to select the study participants from each hospital, the proportional allocation formula was used based on caseloads. After that, a list of medical record numbers of adults diagnosed with DM was prepared from diabetic follow-up logbooks for each hospital. Finally, from the prepared sample frame medical record number lists in each hospital, the computer-generated simple random sampling technique was applied to select the proportionally allocated study participants.

### Study variables

#### Dependent variables

Dependent variables include incidence rate and time to develop HGEs.

#### Independent variables

Independent variables include socio-demographic variables (age, sex, community health insurance, and residence), clinical variables (recent acute illness, type of DM, glycemic control, DM duration, presence of comorbidities, and presence of chronic diabetic complications), and treatment factors (follow-up frequency, type of drug used, and medication non-compliance).

### Operational definitions of variables

#### Survival

Survival is defined as the time measured between the date of DM diagnosis and the date of developing HGEs or the date being censored.

#### Censored

Adults diagnosed with DM who did not develop HGEs during the follow-up study (transfer out, died, lost to follow-up, still not develop HGEs because the study ends) are considered as censored.

#### Adult

Individuals whose age is ≥18 years old are considered adults.

#### Hyperglycemic emergencies

Diabetic ketoacidosis was defined based on a clinical diagnosis of DKA when there is RBGL of > 250 mg/dl, urine ketone body of ≥ +2, arterial pH of < 7.3, and ${\text{HCO}}_{3}^{-}$ of < 15 meq/l. The hyperglycemic hyperosmolar syndrome was considered when RBGL is > 600 mg/dl, with alteration in mental status with minimal or absent urine ketone body (33).

#### Comorbidities

Adults having DM and other chronic medical or psychiatric diseases are considered as comorbidities.

#### Event

The occurrence of HGEs from diagnosis to the end of the study is considered as the event.

#### Incomplete records

Incomplete records are charts with incomplete information for variables of the date of DM diagnosis and the date when the event occurred or the last observation.

#### Glycemic control

The average 3-month FBS level ranges from 70 to 130 mg/dl or HgA1c of < 7% referred to as controlled, and uncontrolled blood glucose is defined as an average FBS of > 130 or < 70 (34).

#### Age categories

Age categories are young (18–44 years old), middle-aged (45–64 years old), and older age (≥ 65 years old) (7).

### Data collection tools and procedures

We developed the data extraction checklist after reviewing different relevant literature (14, 26, 30). Data were collected from patients' charts using a pretested checklist. Charts were retrieved based on their medical registration number in each hospital. The data collection team included chart finders from the chartroom, two BSc nurse data collectors, and one supervising BSc health officer who has previous data collection experience. The extracted data were coded to avoid duplication.

### Data quality assurance

Before the data collection, a pretest was done in Felege Hiwot Comprehensive Specialized Hospital with 24 (5%) of the sample size from charts of adult patients with diabetes that were registered 1 month before the study period and charts were not included in the final sample size. Consequently, necessary modifications were made to the checklist. A 1-day training was given to data collectors and supervisors before actual data collection. The completeness of the collected data was checked on the site daily basis during data collection, with feedback from the supervisor and the investigators. In addition to this, the data were carefully entered and a double data entry was done.

### Data processing and analysis

The data were coded, cleaned, and checked for consistency and completeness, and then the data were entered into EPI data version 4.6.0 and exported into STATA version 14.0 for cleaning, managing, and statistical analysis. Descriptive statistics were presented with frequency tables and graphs for categorical variables, and continuous variables were reported with mean [mean ± standard deviation (SD)] and median [interquartile range (IQR)]. The Kaplan–Meier survival curve was used to estimate the median survival time and the log-rank test was used to statistically test survival between different categories of independent variables. The Cox-proportional hazard model assumption was checked using the Schoenfeld residuals test, the Cox–Snell residual, and the parallel assumption test.

The association between the independent variables and the outcome variable was assessed by the Cox-proportional hazard model. Variables with a *p* < 0.25 in bivariable were a candidate for multivariable analysis. A 95% CI of hazard ratio (AHR) was computed and variables having a *p* < 0.05 in the multivariable model were considered statistically significant with the dependent variables.

## Results

### Demographic characteristics and descriptive statistics

Among the 473 reviewed charts of adult patients with diabetes, 453 of them were included in the study and analyzed. The rest 20(4.2%) charts accounted for incomplete charts and no history of follow-up after their DM diagnosis. Out of 453 adults, half (51.43%) were men and more than half (72.19%) were from urban areas (Table 1). The median age of the patients at the time of diabetes diagnosis was 47 (IQR = 20) years.

**Table 1:** Baseline socio-demographic results of adult patients with diabetes in Bahir Dar public hospitals, North West Ethiopia, 2021 (*n* = 453).

**Variables**	**Category**	**Frequency (*n*)**	**Percent (%)**
Age	Young age	195	43.05
	Middle age	201	44.37
	Older age	57	12.58
Sex	Male	233	51.43
	Female	220	48.57
Residence	Urban	327	72.19
	Rural	126	27.81
Community health insurance	Yes	209	46.14
	No	244	53.86

The majority of patients (317, 69.98%) were patients with T2DM. Out of the total patients with diabetes, 301 (66.45%) had comorbidity and 52 (21.1%) had more than one comorbidity. Cardiovascular disorders, 202(44.59%) were found to be the most common comorbid diseases. Approximately 213 (47.02%) patients had poor 6-month glycemic control (Table 2). Most of the patients, 279 (61.59%), used oral hypoglycemic agents. Approximately 80 (17.66%) adult patients with diabetes had medication non-compliance (Table 3).

**Table 2:** Clinical-related results of adult patients with diabetes in Bahir Dar public hospitals, North West Ethiopia, 2021 (*n* = 453).

**Variables**	**Category**	**Frequency (*n*)**	**Percent (%)**
DM type	T2DM	317	69.98
	T1DM	136	30.02
DM duration category	< 3 years	258	56.95
	≥3 years	195	43.05
Recent six-month glycemic control	Controlled	240	52.98
	Uncontrolled	213	47.02
Acute recent illness	Acute febrile illness	32	7.06
	Respiratory infection	32	7.06
	Urinary tract infection	35	7.73
	Gastrointestinal infection	32	7.06
	Myocardial infarction	7	1.55
	Others^*^	21	4.64
Comorbidities	Chronic respiratory disorder	23	5.08
	Cardiovascular disorder	202	44.59
	HIV/AIDS	25	5.52
	psychiatric disorder	14	3.09
	Renal disease	24	5.30
	Others^**^	13	2.87
	More than one comorbidity	52	21.1
Chronic diabetic complications	Diabetic foot ulcer	22	4.86
	Diabetic neuropathy	26	5.74
	Diabetic nephropathy	18	3.97
	Diabetic retinopathy	9	1.99

^*^Others include meningitis, cellulitis, and thyrotoxicosis.

^**^Others include chronic liver disease, goiter, osteoporosis, dyslipidemia, and benign prostate hyperplasia.

**Table 3:** Treatment-related results of adult patients with diabetes in Bahir Dar public hospitals, North West Ethiopia, 2021 (*n* = 453).

**Variables**	**Categories**	**Frequency (*n*)**	**Percent (%)**
Drug type	Oral hypoglycemic agent	279	61.59
	Insulin	151	33.34
	Oral and insulin	23	5.08
Medication non-compliance	No	373	82.34
	Yes	80	17.66
Follow up frequency	Weekly	28	6.18
	Monthly	192	42.38
	2–3 months	152	33.55
	≥4 months	20	4.42
	Irregular	61	13.47

### Incidence of HGEs among adult patients with diabetes

In total, 453 patients were followed for different periods of 5 years with 12,048 person-month observations. The overall mean follow-up length was 26.6 months (95% CI: 24.97–28.22). Out of the total patients included in the analysis, approximately 147 (32.45%) developed HGEs, of which, 92 (62.59%) were men and 108 (73.47%) had comorbidity. The overall HGEs incidence in the cohort was 14.6 per 100 person-years observation (95% CI, 12.5–17.2). The incidence of DKA was 12.5 per 100 person-years (95% CI, 10.5–14.9), which was 35.6 and 6.3 per 100 person-years among T1DM and T2DM, respectively. The incidence of HHS was 2.1 per 100 person-years (95% CI, 1.4–3.2), which was 0.9 and 2.4 per 100 person-years among T1DM and T2DM, correspondingly. In the entire follow-up period, a higher incidence of HGEs occurred between 0 and 12 months of follow-up interval (Supplementary Table S1).

### Overall survival function

**Figure 1:**
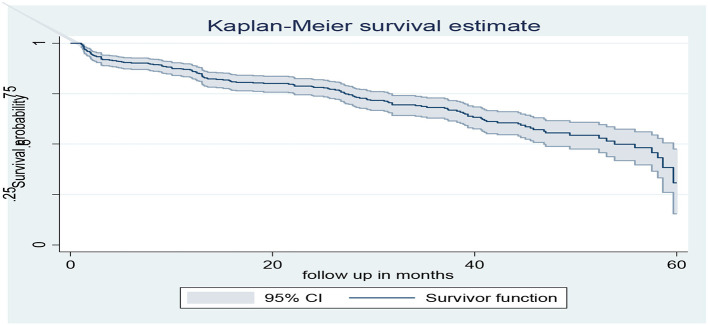
The overall Kaplan–Meier survival curve of HGEs-free survival estimate of adult patients with diabetes in Bahir Dar public hospitals, North West Ethiopia, 2021 (*n* = 453).

The overall Kaplan–Meier estimate showed that the probability of HGEs-free survival of adult patients with diabetes was high during 1.02 months of observation (99.78%). The probability of HGEs-free survival relatively falls as the follow-up month increases, with a sharp fall after 58.62 months, and a minimum HGEs-free survival probability was observed (30.72%) at 60 months of observation (Figure 1). The overall median HGE-free survival time of the entire cohort was 53.85 months (95% CI, 46.88–59.67).

### Survival function and comparison of different categorical variables

The log-rank test was used to check statistically for the presence of any significant differences in survival among various categorical predictors. Patients with controlled six-month glycemic control, with community health insurance, without a history of medication noncompliance, with DM duration ≥ 3 years, without comorbidities, without a history of recent acute illness, and diagnosed with T2DM had longer HGEs-free survival time than the corresponding categories with (Log-rank test, *p*-value ≤ 0.01).

### Bivariable and multivariable Cox regression analysis

In the bivariable Cox Regression model, variables with a *P* < 0.25 were candidates for the multivariable Cox regression analysis. A total of 14 variables fitted the multivariable Cox regression model and eight variables were significant.

In the multivariable Cox regression model, community health insurance, type of DM, DM duration, recent 6-month glycemic control, history of recent acute illness, presence of comorbidities, follow-up frequency of 2–3 months, and history of medication non-compliance were significantly associated with the incidence and time to develop HGEs (*P* < 0.05). Baseline age, sex, duration of treatment started, residency, type of drug used, and chronic diabetic complications were not statistically significant predictors associated with the incidence and time to develop HGEs (Table 4).

**Table 4:** Bivariate and multivariate Cox regression analysis of adult patients with diabetes in Bahir Dar public hospitals, North West Ethiopia, 2021 (*n* = 453).

**Variables**	**Category**	**Censored**	**Developed HGEs**	**CHR [95% CI]**	**AHR [95%CI]**	***P*-value**
Age	Young age	105 (53.5)	90 (46.15)	1		
	Middle age	165 (82.9)	36 (17.91)	0.33 (0.22, 0.48)	0.70 (0.44, 1.13)	0.145
	Older age	36 (63.16)	21 (36.84)	0.65 (0.41, 1.05)	1.04 (0.58, 1.86)	0.895
Sex	Male	141 (60.2)	92 (39.48)	1		
	Female	165 (75.0)	55 (25.00)	0.52 (0.37, 0.73)	0.74 (0.51, 1.06)	0.101
Residence	Urban	253 (77.7)	74 (22.63)	1		
	Rural	53 (42.06)	73 (57.94)	4.39 (3.14, 6.1)	0.94 (0.61, 1.46)	0.784
Community health insurance	Yes	158 (75.0)	51 (24.40)	1		
	No	148 (60.6)	96 (39.34)	1.69 (1.20, 2.37)	1.63 (1.14, 2.35)	0.008
DM type	T2DM	248 (78.3)	69 (21.77)	1		
	T1DM	58 (42.65)	78 (57.35)	4.52 (3.24, 6.3)	2.75 (1.68, 4.51)	< 0.001
Type of drug used	OHGA	228 (81.2)	51 (18.28)	1		
	Insulin	65 (43.05)	86 (56.95)	4.56 (3.22, 6.48)	1.44 (0.89, 2.33)	0.136
	Insulin +OHGA	13 (56.52)	10 (43.48)	2.83 (1.44, 5.58)	1.54 (0.75, 3.19)	0.243
Treatment started	Immediate	297 (68.5)	136 (31.4)	1		
	Not Immediate	9 (45.00)	11 (55.00)	1.98 (1.07, 3.67)	0.93 (0.44, 1.97)	0.858
Follow up frequency	Monthly	163 (84.9)	29 (15.10)	1		
	Weekly	19 (67.86)	9 (32.14)	1.92 (0.89, 4.14)	1.71 (0.72, 4.05)	0.221
	2–3 months	73 (48.03)	79 (51.97)	4.42 (2.89, 6.78)	1.79 (1.06, 3.01)	0.029
	>/=4 mons	3 (15.00)	17 (85.00)	10.23 (5.58, 18.1)	2.02 (0.99, 4.12)	0.053
	Irregular	48 (78.69)	13 (21.31)	1.19 (0.62, 2.30)	1.15 (0.55, 2.40)	0.707
Medication non-compliance	No	284 (76.1)	89 (23.86)	1		
	Yes	22 (27.50)	58 (72.50)	4.50 (3.20, 6.32)	1.85 (1.24, 2.76)	0.003
DM duration	< 3 years	181 (70.1)	77 (29.84)	1		
	≥3 years	125 (64.1)	70 (35.90)	0.33 (0.22, 0.49)	0.33 (0.21, 0.50)	< 0.001
Six-month glycemic control	Controlled	213 (90.6)	22 (9.36)	1		
	Uncontrolled	93 (42.66)	125 (57.3)	6.39 (4.20, 9.71)	3.47 (2.17, 5.56)	< 0.001
Recent acute illness	No	255 (80.9)	60 (19.05)	1		
	Yes	51 (36.96)	87 (63.04)	4.68 (3.36, 6.53)	3.0 (2.03, 4.43)	< 0.001
Comorbidities	No	167 (81.7)	39 (18.93)	1		
	Yes	139 (56.8)	108 (43.2)	2.73 (1.89, 3.95)	2.36 (1.53, 3.63)	< 0.001
Chronic diabetic complications	No	283 (73.2)	103 (26.8)	1		
	Yes	23 (34.33)	44 (65.67)	3.181 (2.23, 4.53)	1.23 (0.81, 1.88)	0.329

CHR, crude hazard rate; AHR, adjusted hazard rate; HR, 1 is a reference category; OHGA, Oral hypoglycemic agent.

## Model checking

### Test of the assumption of proportional hazards

The Schoenfeld residual proportional hazard assumption test for individual covariates and global tests were used. Finally, each covariate had a *P* > 0.05, and the entire covariate combined or global test had a *P*-value of 0.703, all showing a fitted Cox-proportional hazard model.

### Graphically test of proportional hazard assumption

The covariates included in the model were assessed on a plot of log minus log and we observed that the variables included in the final model yielded parallel curves, which showed proportional hazard across the groups.

### Diagnosis of the model

**Figure 2:**
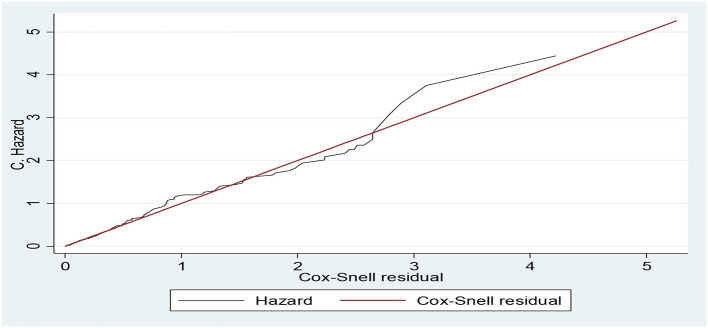
Cumulative hazard vs. Cox–Snell residual plots for Cox regression model diagnosis of the data of adult patients with diabetes in Bahir Dar public hospitals, North West Ethiopia, 2021 (*n* = 453).

The Cox–Snell residual plot shows the overall model fit. The closing line of the Cox–Snell residual vs. the 45-degree bisector of the cumulative hazard line shows the better-fitted model for the data. Our Cox–Snell residual test showed that the model fitted to the data as observed that the Cox residual line was close to the bisector line without strike deviation (Figure 2).

## Discussion

In resource-limited countries, identifying the incidence and predictors of HGEs among patients with diabetes is important for its prevention and management. In this study, the proportion who developed HGEs during the study period was 32.45%. This finding was lower than that of a study done in Hawassa, Ethiopia, 44.6% (29), and greater than the study in Gurage zone hospitals, Ethiopia, 12.7% (35). This discrepancy might be due to previous studies being cross-sectional, which included patients who developed HGEs at the onset of DM diagnosis, but this study did not include them to increase the precision of the survival time of the study. Another plausible reason for the increased HGEs could be that this study was a long follow-up study, but the counter-study was cross-sectional. In addition, this result was much higher than an institution-based retrospective study done in Thailand, 7.46% (20). This difference could be explained by the difference in the study population, lifestyle, culture, level of education, race, environment, and diabetic care services and treatment modalities, which all affect glycemic control and diabetic management (36); consequently, these could affect the incidence of HGEs.

The incidence of HGEs was 14.6 per 100 person-year observation, and this is higher than the study in Korea (2.15 per 1000 PY) (22). The incidence of DKA was 12.5 per 100 person-years. Whereas, its incidence among T1DM and T2DM was 35.6 and 6.3 per 100 person-years, respectively, which is higher than a systematic study conducted in developed countries (0–263 per 1000 PY) (18) and in Northern Sweden (5.9 per 100,00 per year) (37). The incidence of HHS was 2.1 per 100 person-years, while the incidence of HHS among T1DM and T2DM was 0.9 and 2.4 per 100 person-years, correspondingly. This is in line with (19) and higher than the national report in the United States (0.9 per 1000 PY) (7). This higher incidence of DKA and HHS could be due to the counter study being a nationwide study and our study was an institution-based study. The other possible reason might be disparities in economic status, level of education, and access to healthcare facilities between the previous and our general population. Studies done in our study population showed the presence of poor access to healthcare facilities (38), lack of diabetes education, and poor self-care practice (39–41), which may have contributed to the increased incidence of HGEs. Thus, this increased incidence will increase diabetic-related morbidity, premature mortality, and associated costs, as the study (42) showed that HGEs are the major causes of diabetic morbidity, mortality, and increased healthcare expenditure in Ethiopia.

In this study, patients who had no community health insurance had a 1.63 times higher hazard of developing HGEs than those who had community health insurance. This agrees with another study (9). The reason might be that patients with no community health insurance were less likely to afford to perform regular self-blood glucose monitoring and other follow-up examination expenses (43).

Patients with a history of recent acute illness were 2.99 times more hazardous to develop HGEs compared to those without a history of recent acute illness. This is in line with studies in Ethiopia (14, 30) and Iraq (31). The reason might be that acute illness is associated with a hypermetabolic state due to the release of cytokines and counter-regulatory hormones (1), which could induce insulin resistance and increase hepatic glucose production, contributing to the development of HGEs (44).

According to this study, diabetic patients with comorbidities of psychiatric and chronic medical disorders had 2.36 times more hazard of developing HGEs compared to diabetic patients without these issues. This is supported by other studies in Ethiopia (14, 29, 30), multicenter analysis conducted in four countries (Germany, Australia, Switzerland, and Luxemburg) (45), and the USA (27). This might be because the presence of comorbidities and related treatment medications can directly or indirectly disturb the normal functions of insulin and other counter-regulatory hormones. In addition, diabetic patients with chronic medical comorbidities have poor glycemic control (46) and psychiatric comorbidities can compromise adherence to the treatment (47).

In terms of diabetic duration, patients diagnosed with diabetes greater and equal to 3 years of duration had 67% less hazard of developing HGEs compared to patients with diabetes of shorter duration. Another study in Iraq (31) also showed that diabetic duration was a statistically significant predictor. Conversely, a study in Ethiopia (32) revealed that diabetic duration was not statistically significant. This inconsistency could be due to methodology and clinical characteristics differences. For example, the counter study was a case–control study and specific to DKA, while this study included HHS. The counter study also had patients with newly diagnosed DM and patients with DKA, who are excluded from our study. Furthermore, studies showed that patients with diabetes during the early years of diagnosis had stress and anxiety about the disease and had less knowledge and practice about the prevention of acute diabetic complications (48). Therefore, the decreased hazard of developing HGEs during 3 years duration and longer could reveal that as the diabetic duration increases, patients will get adequate knowledge about the disease and prevention of HGEs.

Regarding the preceding 6-month glycemic control, this study noted that poor glycemic control was a strong predictor of HGEs and this is comparable with studies in the USA (49) and Australia (50). Adult patients with diabetes with poor 6-month glycemic control had 3.47 times more hazards of developing HGEs compared to patients with controlled glycemic control. The reason might be explained that both lower and higher blood sugar levels from the normal range can lead to a point spectrum of hyperglycemic emergencies by increasing counter-regulatory hormones, altered glucose production, and lipolysis (1).

Our study revealed that adult patients with T1DM had 2.75 times more hazards to develop HGEs than adult patients with T2DM. This finding is supported by other studies in Ethiopia (30, 32). This could be theorized that DKA is the more frequent HGE and is common in T1DM, which is due to that patients with type 1 diabetes had a deficiency of insulin to suppress lipolysis in different conditions, resulting in ketone formation (1).

The history of medication non-compliance was a significant predictor of HGEs. Other research conducted in Ethiopia showed a similar result (AOR 4.31 95% CI, 1.92–9.68) (32). The risk of developing HGEs among patients who had a history of medication non-compliance was 1.85 times more hazardous relative to those who are without a history of medication non-compliance. This is because not taking or not following the prescribed course of antidiabetic medication treatment could increase the blood glucose level which is the major cause of HGEs (1), and the lower magnitude of risk of developing HGEs in our study could be the result of under documentation as our study was secondary data.

Concerning diabetic follow-up frequency, patients who had a diabetic follow-up every 2–3 months are 1.79 times more hazardous to develop HGEs than those who had a diabetic follow-up every month. Another study (32) also showed that patients with diabetes with regular follow-up visits had a lower risk of developing HGEs. This might be due to having shorter diabetic care follow-up visits that will allow healthcare providers to ensure that patients are moving forward with the recommended self-care and medication use, and to give education about the prevention of HGEs and modifiable risk factors.

Finally, different studies in the world showed that age (27, 31), sex (29, 31), and residence (28, 31) were significantly associated with HGEs, and conversely, other studies showed that age (51), sex (27, 28), and residence (30) were not the significant predictors of HGEs. Similarly, our findings showed that these three variables are not statistically associated with HGEs. The variation of our results from others might be due to differences in research methodology, race, residence, socio-cultural, lifestyle, treatment, and diagnosis modality differences. Another possible reason could be that since our data was secondary, there could be under documentation.

## Conclusion and recommendations

The incidence of HGEs among adult patients with diabetes was found to be high compared to previous studies. The incidence was higher and predicted for those patients who had type 1 diabetes mellitus, a history of medication non-compliance, comorbidities, recent acute illness, poor glycemic control, follow-up frequency of 2–3 months, and who are without community health insurance. On the contrary, patients diagnosed with diabetes greater and equal to 3 years of duration had less hazard of developing HGEs. As a result, we recommend healthcare providers to monitor thoroughly and have close follow-ups for patients with the identified predictors and should also give adequate knowledge about HGEs and prevention practices for newly diagnosed patients. In addition, including patients with diabetes in community-based health insurance and making a diabetic follow-up within a month could decrease the incidence and related public health and economic impacts. Finally, we recommend conducting further prospective studies to address the missed variables and recurrent HGEs should be investigated with another study design, as this was not assessed in our study.

## Data availability statement

The raw data supporting the conclusions of this article will be made available by the authors, without undue reservation.

## Ethics statement

The studies involving human participants were reviewed and approved by Institutional Review Board (IRB) of Bahir Dar University's Ethical Review Committee. Written informed consent for participation was not required for this study in accordance with the national legislation and the institutional requirements. Written informed consent was not obtained from the individual(s) for the publication of any potentially identifiable images or data included in this article. No potentially identifiable human images or data are presented in the manuscript.

## Author contributions

All authors have made substantial contributions to the study reported, whether that is in the conception of this study, design of the study, acquisition, analysis, and interpretation of data, or all these areas, took part in drafting, revising, or critically reviewing the article, read and gave the final approval for the version to be published, agreed to the journal to which the article has been submitted, and agree to be accountable for all aspects of the study.
