# Supplementary material for: Incidence and predictors of hyperglycemic emergencies among adult diabetic patients in Bahir Dar city public hospitals, Northwest Ethiopia, 2021: A multicenter retrospective follow-up study
Source: Front Public Health. 2023 Mar 17;11:1116713. doi: 10.3389/fpubh.2023.1116713 (PMC10063795; doi:10.3389/fpubh.2023.1116713)
Supplement: Supplementary file 1 [file Table_1.DOCX]

Table S1: Life table showing the survival to develop HGEs among adult diabetic patients in Bahir-Dar Public Hospitals, North West Ethiopia, 2021(n=453)

| Interval  In months | Patients at risk | No of HGEs cases | Censored | Cumulative Survival | SD. Error | [95% CI] | |
| --- | --- | --- | --- | --- | --- | --- | --- |
|  |  |  |  |  |  | L | U |
|  |  |  |  |  |  |  |  |
| [0 12] | 453 57 57 0.8657 0.0165 0.8295 0.8948 | | | | | | |
| ( 12 24] | 339 30 64 0.7811 0.0209 0.7367 0.8190 | | | | | | |
| (24 36] | 245 29 60 0.6758 0.0257 0.6226 0.7232 | | | | | | |
| (36 48] | 156 22 76 0.5498 0.0320 0.4849 0.6099 | | | | | | |
| (48 60] | 58 9 47 0.4064 0.0474 0.3132 0.4973 | | | | | | |
| ( 60 72] | 2 0 2 0.4064 0.0474 0.3132 0.4973 | | | | | | |
